# Combination of two anti-tubulin agents, eribulin and paclitaxel, enhances anti-tumor effects on triple-negative breast cancer through mesenchymal-epithelial transition

**DOI:** 10.18632/oncotarget.25184

**Published:** 2018-05-01

**Authors:** Takaaki Oba, Ken-Ichi Ito

**Affiliations:** ^1^ Division of Breast, Endocrine and Respiratory Surgery, Department of Surgery II, Shinshu University School of Medicine, 3-1-1 Asahi, Matsumoto, Japan

**Keywords:** eribulin, paclitaxel, triple-negative breast cancer, epithelial-mesenchymal transition, mesenchymal-epithelial transition

## Abstract

Improved prognosis for triple-negative breast cancer (TNBC) has currently plateaued and the development of novel therapeutic strategies is required. Therefore, we aimed to explore the anti-tumor effect of eribulin and paclitaxel combination therapy for TNBC. The effect of eribulin and paclitaxel in combination was tested, with both concurrent and sequential administration, using four TNBC cell lines (MDA-MB-231, Hs578T, MDA-MB-157, and Mx-1) *in vitro* and in an MDA-MB-231 BALB/c-nu/nu mouse xenograft model. The expression of epithelial-mesenchymal phenotypic markers was analyzed by western blotting and immunohistochemical analyses. Simultaneous administration of eribulin and paclitaxel resulted in a synergistic anti-tumor effect with MDA-MB-231 and Hs578T cells, but not MDA-MB-157 and Mx-1 cells, *in vitro*. Moreover, pre-treatment with one drug significantly enhanced sensitivity to the subsequently administrated second drug in MDA-MB-231 and Hs578T cells. Eribulin increased E-cadherin expression and decreased the expression of mesenchymal markers in MDA-MB-231 and Hs578T cells. In contrast, paclitaxel increased the expression of mesenchymal markers. When epithelial-mesenchymal transition was induced by TGF-β1, eribulin sensitivity was enhanced. In contrast, a TGF-β receptor kinase inhibitor decreased eribulin sensitivity. In MDA-MB-231 tumor-bearing mice, concurrent administration of low doses of eribulin and paclitaxel significantly inhibited tumor growth compared to that with either monotherapy. Moreover, single administration of eribulin before the initiation of paclitaxel treatment decreased vimentin expression and reduced the average tumor volume in a mouse xenograft model. Eribulin and paclitaxel show synergistic anti-tumor effect by altering the epithelial-mesenchymal phenotype. This combination therapy could represent a novel therapeutic strategy for TNBC.

## INTRODUCTION

Triple-negative breast cancer (TNBC) is a disease characterized by the lack of estrogen receptor (ER) and progesterone receptor (PgR) expression as well as human epidermal growth factor receptor 2 (HER2) amplification, and accounts for 10–20% of all breast cancers. Recent progress in targeted therapies, both in adjuvant and metastatic settings, has improved the prognosis of ER-positive and HER2-positive breast cancers, whereas the improvement in survival has currently plateaued for TNBC [1]. Although conventional cytotoxic chemotherapy based on anthracyclines and taxanes is effective for a subset of patients with TNBC, some cases show a very aggressive clinical course. TNBC is associated with a higher rate of distant recurrence and a poorer prognosis than those in patients with other breast cancer subtypes, and fewer than 30% of patients with metastatic TNBC survive five years [2–5]. Hence, there is an immediate need to develop novel therapeutic strategies for TNBC.

Microtubules are important structural and functional components of cells, and they represent an important therapeutic target of anti-cancer drugs [6]. Several anti-tubulin agents such as paclitaxel, docetaxel, vinorelbine, and epothilone have been used to treat breast cancer [7]. Recently, eribulin mesylate (eribulin) was introduced for the treatment of metastatic breast cancer. This compound is a synthetic macrocyclic ketone analog of halichondrin B, naturally occurring in marine sponges, and inhibits microtubule polymerization via a mechanism distinct from that of other anti-tubulin agents such as vinblastine or taxanes [8, 9]. When administered to patients with metastatic breast cancer who had previously received both anthracycline and taxane, eribulin alone significantly increased overall survival (OS) [10]. Consequently, this drug is currently used for patients with advanced breast cancer. One unique characteristic of eribulin, which emerged from the results of two phase 3 clinical trials for metastatic breast cancer, is that it has more pronounced effects on OS than progression-free survival (PFS) [10, 11]; however, the underlying mechanisms of this phenomenon are not understood.

Epithelial-mesenchymal transition (EMT), and its reverse process mesenchymal-epithelial transition (MET), were originally identified in early embryonic development [12]. Transforming growth factor-β (TGF-β) is known as the primary inducer of EMT [13, 14]. Several studies have demonstrated that this process correlates with tumor progression, metastasis, and development of drug resistance [15, 16].

Recent studies have reported that paclitaxel-resistant TNBC cells have mesenchymal characteristics such as higher expression of mesenchymal markers like vimentin and N-cadherin. As such, inhibition of TGF-β signaling, with TGF-β type I receptor kinase inhibitors, attenuates metastasis after paclitaxel therapy and enhances the anti-tumor effect of this chemotherapeutic [17, 18]. Thus, combination therapy with paclitaxel and TGF-β receptor kinase inhibitors represents a potential therapeutic strategy for TNBC.

In contrast to paclitaxel, recent studies have demonstrated that eribulin can trigger a phenotypic shift from mesenchymal to epithelial phenotypes in TNBC, soft tissue sarcoma, and oral squamous cell carcinoma, in addition to its anti-cancer mechanisms associated with classical antimitotic effects [19–21]. Considering the effects of these tubulin-targeting agents, we hypothesized that the induction of MET by eribulin might enhance the anti-tumor effect of paclitaxel in TNBC. In this study, we demonstrate a synergistic anti-tumor effect by combining eribulin with paclitaxel and determined that this occurred through their opposing effects on the EMT-MET axis both *in vitro* and *in vivo*.

## RESULTS

### Synergistic growth inhibitory effect of eribulin and paclitaxel on TNBC cells *in vitro*

**Figure 1:**
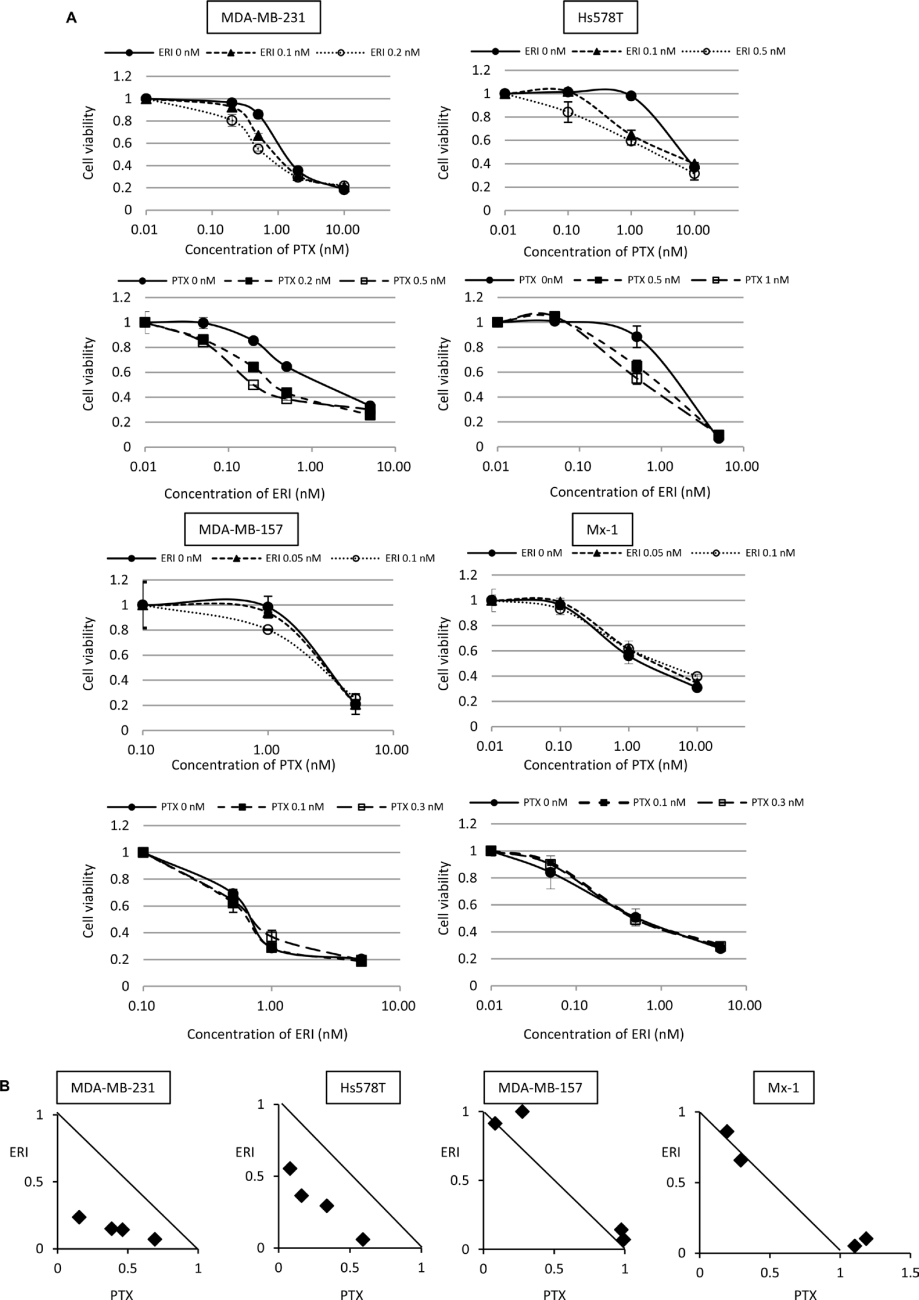
Combinatory effect of eribulin and paclitaxel on growth inhibition of triple-negative breast cancer (TNBC) cells *in vitro* The combinatory effect of eribulin (ERI) and paclitaxel (PTX) on MDA-MB-231, Hs578T, MDA-MB-157, and Mx-1 cells was tested using WST assays. (**A**) Sensitivity to PTX in the presence or absence of low doses of ERI (upper panels for each cell line) and sensitivity to ERI in the presence or absence of low doses of PTX (lower panels for each cell line). Closed circles (●) indicate control, closed triangles (▲) indicate 0.1 nM (MDA-MB-231 and Hs578T cells) or 0.05 nM (MDA-MB-157 and Mx-1 cells) of ERI, open circles (○) indicate 0.1 nM (MDA-MB-157 and Mx-1 cells), 0.2 nM (MDA-MB-231 cells), or 0.5 nM (Hs578T cells) of ERI, closed squares (■) indicate 0.1 nM (MDA-MB-157 and Mx-1 cells), 0.2 nM (MDA-MB-231 cells), or 0.5 nM (Hs578T cells) of PTX, and open squares (□) indicate 0.3 nM (MDA-MB-157 and Mx-1 cells), 0.5 nM (MDA-MB-231 cells), or 1.0 nM (Hs578T cells) of PTX. The error bars represent the standard deviations of the values obtained; experiments were performed in triplicate. (**B**) The experimental data were plotted on an isobologram. The dots located below, on, or above the diagonal line indicate synergistic, additive, and antagonistic effects, respectively.

We investigated the potential augmented growth inhibitory effects of eribulin with paclitaxel in TNBC cells *in vitro*. Here, MDA-MB-231, Hs578T, MDA-MB-157, and Mx-1 cells were simultaneously treated with these agents for 96 h and cell viability was measured using WST assays. Before this experiment, the growth inhibitory effect of monotherapy was tested in these cell lines, and the concentrations at which cell growth was not inhibited were determined for each drug and each cell line (Supplementary Figure 1 and Supplementary Table 1). Subsequently, sufficiently low concentrations of eribulin or paclitaxel, as determined in these preliminary experiments, were used for further experiments. The growth inhibitory effect of paclitaxel was enhanced when low concentrations (0.1 nM or 0.2 nM) of eribulin were simultaneously added to MDA-MB-231 cells. Furthermore, the growth inhibitory effect of eribulin was enhanced when low concentrations (0.2 nM or 0.5 nM) of paclitaxel were simultaneously added to MDA-MB-231 cells. A similar enhanced growth inhibitory effect was observed in Hs578T cells with low concentrations of eribulin or paclitaxel. However, in MDA-MB-157 and Mx-1 cells, a low dose of eribulin did not enhance sensitivity to paclitaxel and paclitaxel did not enhance the sensitivity to eribulin (Figure 1A). During isobologram analysis, each experimental data point was located below the diagonal line for MDA-MB-231 cells and Hs578T cells, indicating that eribulin and paclitaxel acted synergistically. However, for MDA-MB-157 and Mx-1 cells, each experimental data point was typically located on the diagonal line, indicating that growth inhibitory effects of paclitaxel and eribulin were additive (Figure 1B).

### Eribulin and paclitaxel have opposite effects on the EMT-MET axis in TNBC cells

**Figure 2:**
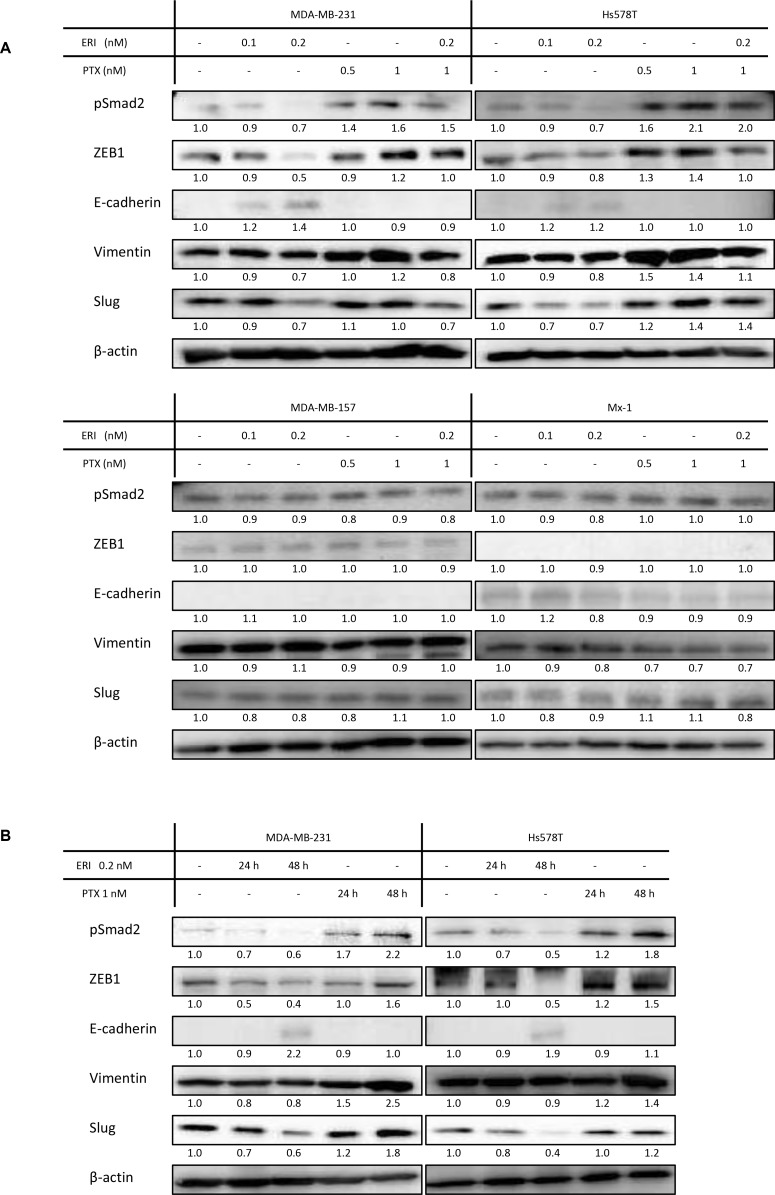
Expression of epithelial/mesenchymal markers in triple-negative breast cancer (TNBC) cell lines The expression of epithelial and mesenchymal markers was studied by western blotting. Representative results of western blot analyses are shown. β-Actin was used as a loading control. The experiments were performed independently at least three times, and one representative blot is provided in the figures. (**A**) Expression of EMT markers in MDA-MB-231, Hs578T, MDA-MB-157, and Mx-1 cells treated with eribulin (ERI; 0.1 and 0.2 nM), paclitaxel (PTX; 0.5 and 1 nM), or both (ERI; 0.2 nM and PTX; 1 nM) for 96 h. (**B**) Expression of EMT markers in MDA-MB-231, Hs578T cells treated with ERI (0.2 nM) or PTX (1 nM) for 24 h and 48 h.

As we found a synergistic effect between eribulin and paclitaxel in MDA-MB-231 and Hs578T cells, but not in MDA-MB-157 and Mx-1 cells, we examined the expression of EMT markers with eribulin or/and paclitaxel treatment. The expression of pSmad2, ZEB1, vimentin, and Slug was studied as mesenchymal markers, whereas that of E-cadherin was studied as an epithelial marker. Western blotting demonstrated that eribulin addition decreased the expression of pSmad2, ZEB1, vimentin, and Slug and increased the expression of E-cadherin in a dose-dependent manner in MDA-MB-231 and Hs578T cells. In contrast, paclitaxel addition increased the expression of pSmad2, ZEB1, vimentin, and Slug in a dose-dependent manner in both cell lines. When the cells were treated with 0.2 nM eribulin and 1 nM paclitaxel simultaneously, the expression of these mesenchymal markers was downregulated compared to that in cells treated with 1 nM paclitaxel alone (Figure 2A, Supplementary Figure 2A). Moreover, the expression of EMT markers increased in a time-dependent manner (Figure 2B, Supplementary Figure 2B). In contrast, the addition of eribulin did not affect the expression of pSmad2, ZEB1, and vimentin in MDA-MB-157 and Mx-1 cells. These results indicate that eribulin and paclitaxel act in an opposing way with regard to the EMT-MET axis in MDA-MB-231 and Hs578T cells, but not in MDA-MB-157 and Mx-1 cells.

### Pre-treatment with eribulin increases paclitaxel sensitivity and vice versa in MDA-MB-231 and Hs578T cells

**Figure 3:**
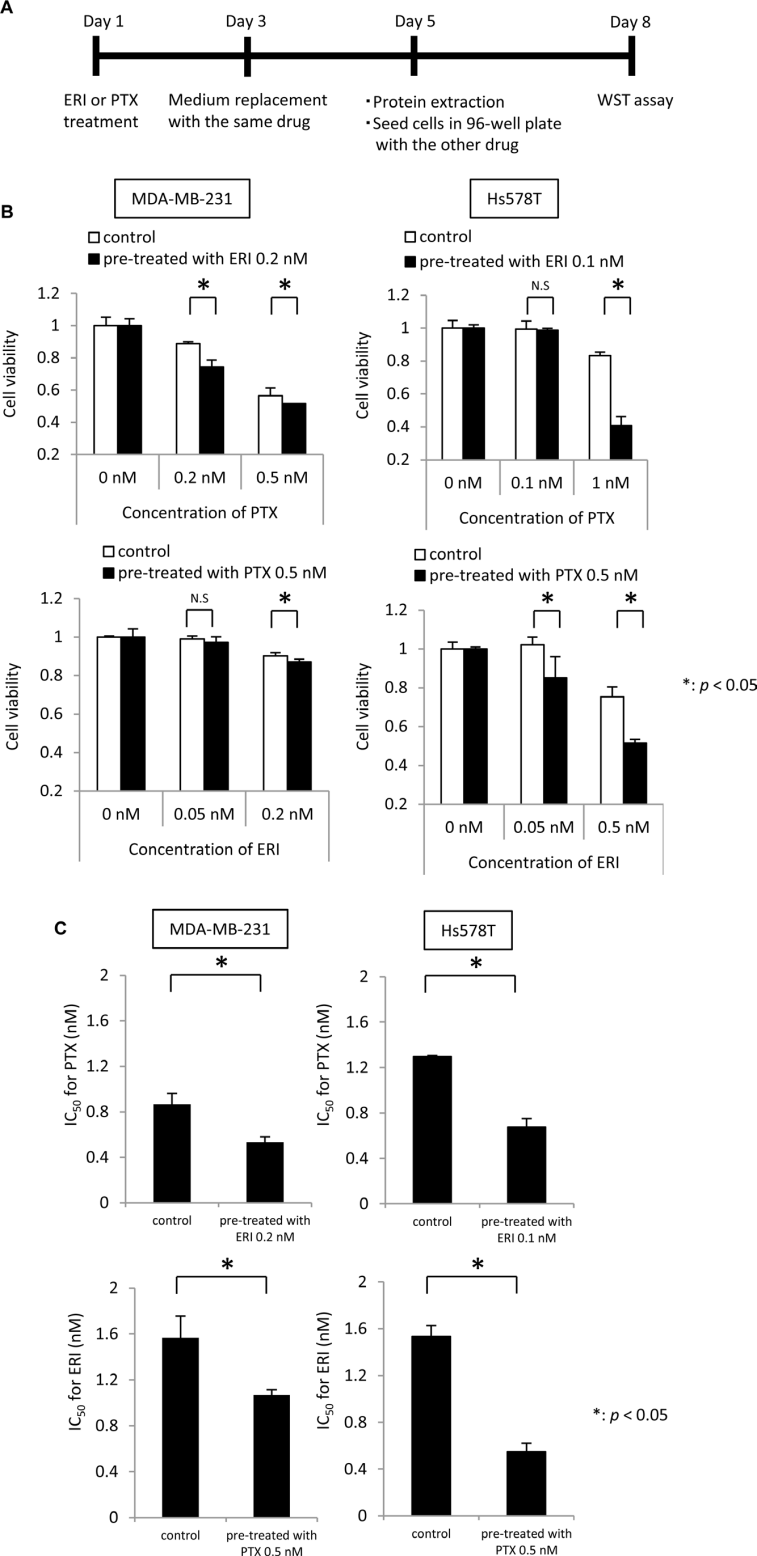
Effects of eribulin pre-treatment on paclitaxel sensitivity and paclitaxel pre-treatment on eribulin sensitivity Sensitivity to paclitaxel (PTX) was tested in cells pre-treated with low doses of eribulin (ERI), and sensitivity to ERI was tested in the cells pre-treated with low doses of PTX, using MDA-MB-231 cells and Hs578T cells. (**A**) Schematic representation of the experiment. (**B**) Sensitivity to 0.2 nM and 0.5 nM of PTX in the MDA-MB-231 cells and 0.1 nM and 1 nM of PTX in the Hs578T cells was measured in cells pre-treated with ERI (0.2 nM for MDA-MB-231 cells, 0.1 nM for Hs578T cells) for 72 h (upper panels for each cell line). Sensitivity to 0.05 nM and 0.2 nM of ERI in the MDA-MB-231 cells and 0.05 nM and 0.5 nM of ERI in the Hs578T cells was measured in the MDA-MB-231 and Hs578T cells pre-treated with 0.5 nM of PTX for 72 h (lower panels for each cell line). White bars indicate the control cells pre-treated with medium alone. Black bars indicate the cells pre-treated with ERI (upper panels) or PTX (lower panels). Error bars represent the standard deviation; the experiments were performed in triplicate. (**C**) IC_50_ for PTX and ERI in cells pre-treated with 0.2 nM ERI in the MDA-MB-231 cells or 0.1 nM ERI in the Hs578T cells (upper panels) or 0.5 nM PTX (lower panel). Cells pre-treated with medium alone were used as a control. IC_50_ values were calculated from the concentration-response curve. Error bars represent standard deviation; experiments were performed in triplicate. ^*^p < 0.05, n.s. = not significant, based on a Student’s *t*-test.

We further analyzed the mechanisms underlying this synergistic effect in MDA-MB-231 and Hs578T cells. To clarify the effects of each drug separately, we examined whether pre-treatment with eribulin or paclitaxel could alter the sensitivity to the other drug subsequently administered *in vitro*. After treating MDA-MB-231 or Hs578T cells with low doses of eribulin (0.2 nM for MDA-MB-231 cells, 0.1 nM for Hs578T cells) or paclitaxel (0.5 nM for both cell lines) from day 1 to day 5 for 96 h, the cells were seeded in a 96-well plate and the pre-treated cells were tested for sensitivity to the other drug (Figure 3A). After incubation for another 72 h, cell viability was measured. Pre-treatment with low doses of eribulin for 96 h significantly enhanced sensitivity to paclitaxel in both cell lines (*p* < 0.05), and pre-treatment with 0.5 nM paclitaxel significantly enhanced sensitivity to eribulin in both cell lines (*p* < 0.05) (Figure 3B). The IC_50_ (half maximal inhibitory concentration) of paclitaxel for the cells pre-treated with control medium was 0.8 nM for MDA-MB-231 and 1.2 nM for Hs578T cells, whereas that for cells pre-treated with 0.1 nM of eribulin was reduced to 0.5 nM for MDA-MB-231 and 0.6 nM for Hs578T cells. The IC_50_ of eribulin for cells pre-treated with control medium was 1.6 nM for MDA-MB-231 and 1.5 nM for Hs578T cells, whereas that for cells pre-treated with 0.5 nM of paclitaxel decreased to 1.0 nM for MDA-MB-231 and 0.5 nM for Hs578T cells. Thus, pre-treatment of these TNBC cells with low doses of eribulin or paclitaxel induced a significant increase in sensitivity to the other anti-tubulin agent (*p* < 0.05; Figure 3C).

### Effect of EMT on sensitivity to eribulin in MDA-MB-231 and Hs578T cells

As we observed that paclitaxel induces EMT and eribulin induces MET in MDA-MB-231 and Hs578T cells, and that pre-treatment with eribulin or paclitaxel increases sensitivity to the other drug, we next examined whether sensitivity to eribulin could be altered by EMT induced by TGF-β.

**Figure 4:**
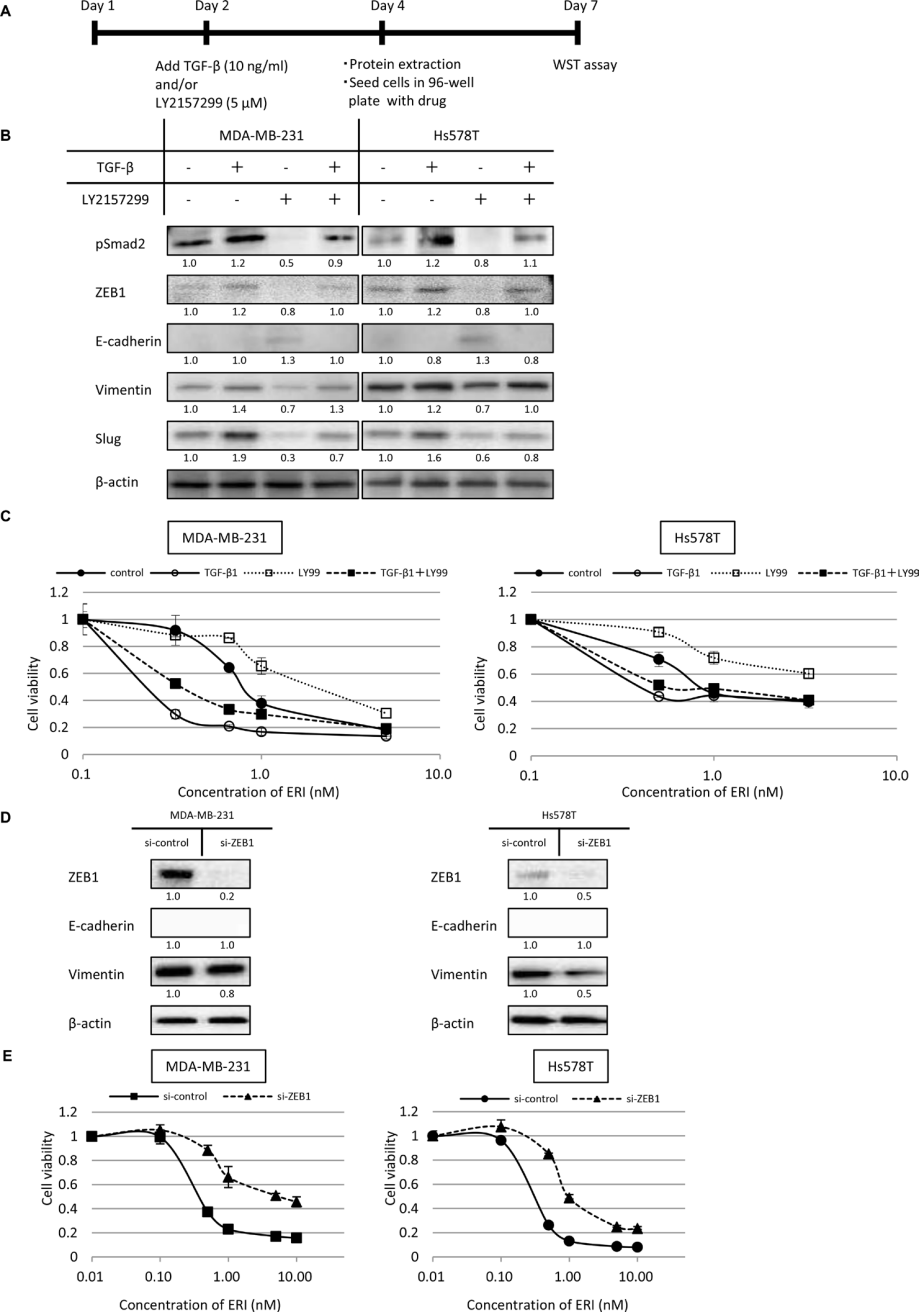
Effects of epithelial-mesenchymal switch on the expression of epithelial/mesenchymal markers and sensitivity to eribulin in MDA-MB-231 and Hs578T cells MDA-MB-231 and Hs578T cells were pre-treated with TGF-β (10 ng/ml) or the TGF-β type I receptor kinase inhibitor LY2157299 (5 µM), or both for 2 days. Subsequently, the expression of epithelial/mesenchymal markers was examined by western blotting. Then, both TGF-β1 and LY2157299 were removed from the media, and eribulin (ERI) sensitivity was tested using WST assays. (**A**) Schematic representation of this experiment. (**B**) Representative results of western blot analyses. β-Actin was used as a loading control. (**C**) Sensitivity to ERI measured using WST assays. Closed circles (●) indicate control, open circles (○) indicate TGF-β, open squares (□) indicate LY2157299 (LY99), and closed squares (■) indicate TGF-β and LY99. Error bars represent standard deviation; experiments were performed in triplicate. The expression of ZEB1 was inhibited by siRNA and sensitivity to eribulin (ERI) was tested using WST assays. The cells were transfected with siRNA targeting ZEB1 (si-ZEB1) or control siRNA (si-control). Twenty-four hours after transfection, protein was extracted and 4 × 10^3^ cells/well were cultured in 96-well tissue culture plates and incubated for 72 h after adding stepwise dilutions of ERI. (**D**) The expression of ZEB1 in the cells was analyzed by western blotting. (**E**) Sensitivity to ERI was measured using WST assays. Closed circles (●) indicate the cells transfected with control siRNA, and closed triangles (▲) indicate the cells with si-ZEB1. Error bars represent standard deviation; experiments were performed in triplicate.

MDA-MB-231 and Hs578T cells were exposed to TGF-β1 (10 ng/ml), the selective TGF-β type I receptor kinase inhibitor, LY2157299 (5 µM), or both for 2 days (Figure 4A). pSmad2, ZEB1, vimentin, and Slug expression increased with the addition of TGF-β but decreased with the addition of LY2157299 in both cell lines. In contrast, E-cadherin expression increased with the addition of LY2157299 in both cell lines. Moreover, increased expression of pSmad2, ZEB1, vimentin, and Slug induced by TGF-β was reversed upon treatment with LY2157299 (Figure 4B, Supplementary Figure 3). These results indicate that TGF-β induces EMT and LY2157299 induces MET in these TNBC cells.

Next, we examined the eribulin sensitivity of cells pre-treated with TGF-β1, LY2157299, or both. Before this experiment, we confirmed that exposure with TGF-β1 (10 ng/ml) or LY2157299 (5 µM) for 2 days did not affect the growth of both cell lines (Supplementary Figure 4). Both MDA-MB-231 and Hs578T cells pre-treated with TGF-β1 were considerably more sensitive to eribulin, whereas MDA-MB-231 and Hs578T cells pre-treated with LY2157299 became resistant to eribulin. Moreover, the increased eribulin sensitivity induced by TGF-β1 was mitigated by the addition of LY2157299, both in MDA-MB-231 and Hs578T cells (Figure 4C). These results indicate that sensitivity to eribulin was enhanced by EMT in these TNBC cells.

### ZEB1 knockdown by siRNA confers resistance to eribulin

To further examine the association between EMT and eribulin sensitivity, we tested whether siRNA-mediated knockdown of ZEB1, which serves as a transcriptional activator of mesenchymal differentiation, could reduce eribulin sensitivity in MDA-MB-231 and Hs578T cells.

Inhibition of ZEB1 expression was confirmed by western blotting (Figure 4D, Supplementary Figure 5). In both cell lines, the expression of E-cadherin was not detected by western blot analysis. However, after ZEB1 knockdown, vimentin expression decreased by half in Hs578T cells and slightly in MDA-MB-231 cells. Compared to MDA-MB-231 and Hs578T cells treated with control siRNA, those treated with siRNA targeting ZEB1 were more resistant to eribulin (Figure 4E). These results indicate that eribulin sensitivity could be decreased when TNBC cells were forced toward an epithelial phenotype.

### The MET phenotype is maintained in eribulin-resistant MDA-MB-231 and Hs578T cells

Our results, together with previous reports [21, 22], demonstrate that eribulin induces MET in TNBC cells after short-term treatment. However, no reports have indicated whether this MET phenotype is maintained in breast cancer cells that have acquired eribulin resistance after long-term exposure. Hence, we examined the expression of EMT-related proteins in the eribulin-resistant MDA-MB-231 and Hs578T cells, which had been established in our laboratory [23].

**Figure 5:**
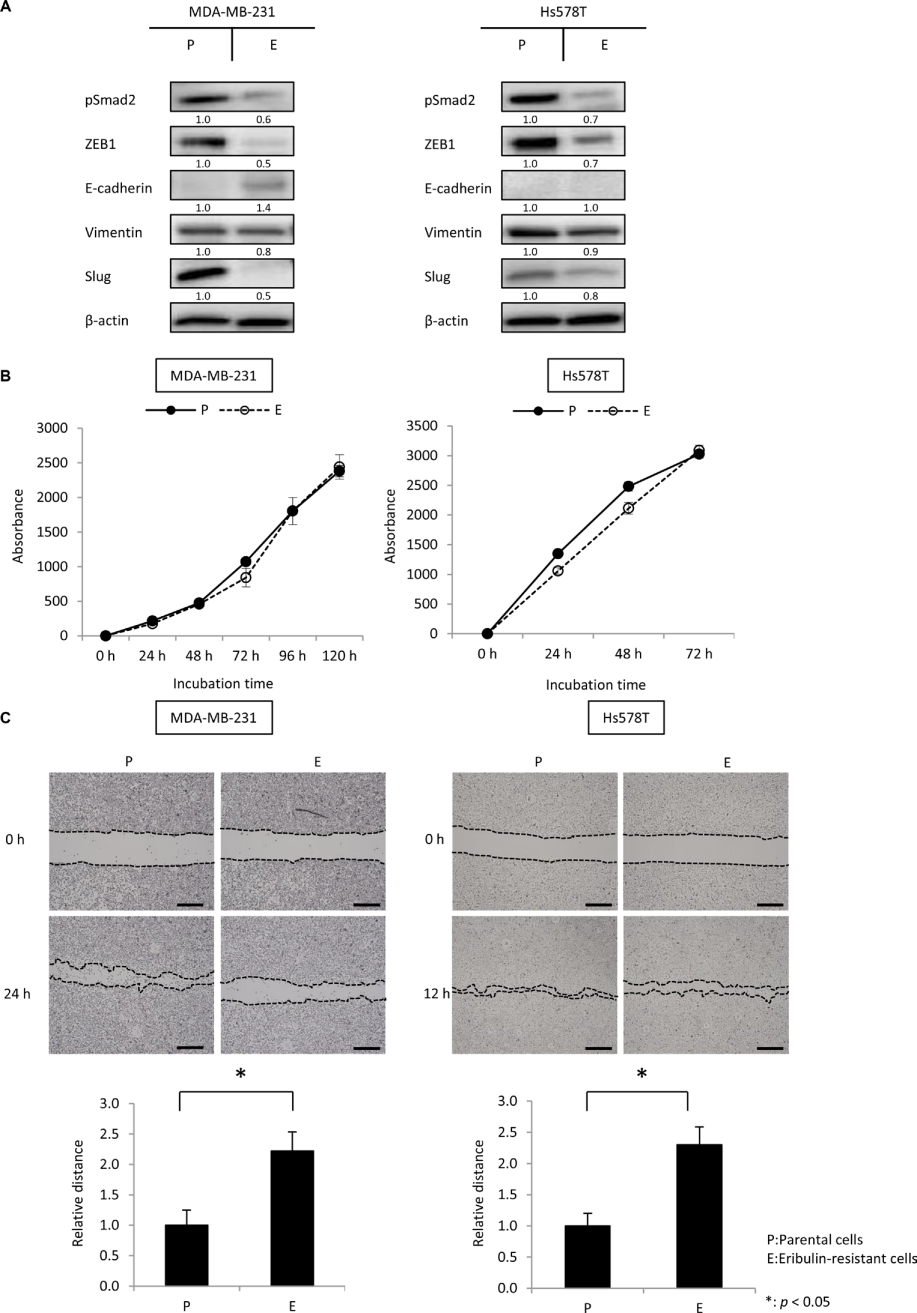
The mesenchymal phenotype was maintained in eribulin-resistant MDA-MB-231 and Hs578T cells The expression of pSmad2, ZEB1, E-cadherin, vimentin, and Slug was analyzed by western blotting using eribulin-resistant MDA-MB-231 and Hs578T cells and the respective parental cells. Cell growth and migration ability were investigated using cell growth and wound healing assays, respectively. (**A**) Representative results of western blot analyses in eribulin-resistant MDA-MB-231 and Hs578T cells (E) and their parental cells (P). β-Actin was used as a loading control. (**B**) Results of cell growth assays performed using eribulin-resistant MDA-MB-231 and Hs578T cells (E) and their parental cells (P). Error bars represent standard deviation; experiments were performed in triplicate. (**C**) Results of wound healing assays performed in eribulin-resistant MDA-MB-231 and Hs578T cells (E) and their parental cells (P). Representative images of wound healing assays after a confluent cell monolayer was scratched at 0 and 24 h for MDA-MB-231 cells and 0 and 12 h for Hs578T cells (upper panels, scale bar = 200 µm). The average wound closure of 100 randomized points was calculated as a ratio of the initial wound size (lower panels). Error bars represent standard deviation based on three independent experiments. ^*^p < 0.05 for parental cells vs. eribulin-resistant cells.

Eribulin-resistant MDA-MB-231 cells (MDA-MB-231/E) and Hs578T cells (Hs578T/E) were 7.9- and 37.7-fold more resistant to EMT, compared to their respective parental cells [23]. pSmad2, ZEB1, vimentin, and Slug levels were remarkably downregulated in eribulin-resistant cells, compared to those in parental cells in both MDA-MB-231 and Hs578T cells. In contrast, the expression of E-cadherin was upregulated in MDA-MB-231/E cells, whereas E-cadherin was not detected in both parental and Hs578T/E cells (Figure 5A, Supplementary Figure 6).

Because cellular migration is known to be associated with an EMT phenotype, we compared the migratory ability of eribulin-resistant cells to that of parental cells by performing wound healing assays. Before the wound healing assay, we examined the proliferative ability of parental and eribulin-resistant cells, and confirmed that this parameter, which could affect the results of the wound healing assay, was not different between eribulin-resistant and parental cells for both MDA-MB-231 and Hs578T cells (Figure 5B). Eribulin-resistant cells showed lower migration ability compared to that of parental cells for both MDA-MB-231 and Hs578T cells (Figure 5C). These results indicate that these TNBC cells maintained an MET phenotype upon acquiring resistance to eribulin.

### Anti-tumor effects of simultaneous administration of eribulin and paclitaxel *in vivo*

**Figure 6:**
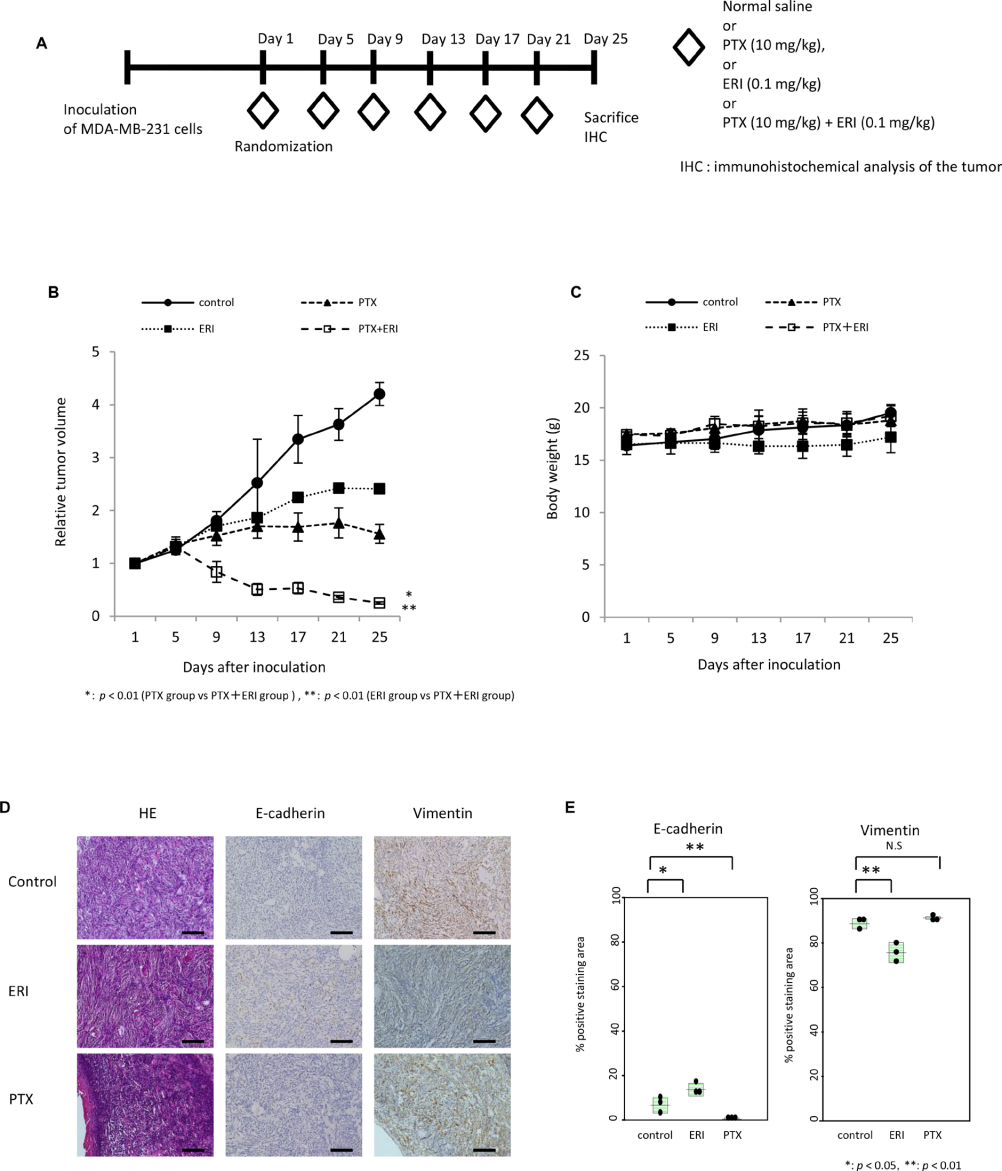
Combinatory effect of eribulin and paclitaxel in mouse xenograft models The effect of combining eribulin (ERI) with paclitaxel (PTX) was tested in an MDA-MB-231 tumor xenograft model. Here, 10 mg/kg of paclitaxel (PTX), 0.1 mg/kg of eribulin (ERI), or both, were intraperitoneally administered every 4 days, six times, and the mice were sacrificed on day 25. (**A**) Schematic representation of the experiment. (**B**) The average relative tumor volumes were plotted from day 1 to day 25, with measurements taken every 4 days. Closed circles (●) indicate control, open circles (○) indicate control, closed triangles (▲) indicate PTX, closed squares (■) indicate ERI, and open squares (□) indicate ERI+PTX. Error bars represent standard deviation. ^*^p < 0.01 (PTX group vs. PTX+ERI group), ^**^p < 0.01 (ERI group vs. PTX+ERI group). (**C**) The average body weight was measured from day 1 to day 25 every 4 days, for each group. (**D**) Representative photographs of HE staining and immunohistochemistry (×200) for E-cadherin and vimentin in tumors obtained from control, PTX, and ERI groups at day 25. Scale bars = 100 µm. (**E**) Comparison of the quantitative data of the positive staining areas for E-cadherin or vimentin observed in the three groups.

Next, we tested the effect of combination treatment of eribulin and paclitaxel in an MDA-MB-231 tumor xenograft model *in vivo.* Paclitaxel (10 mg/kg), eribulin (0.1 mg/kg), or both were intraperitoneally administered to mice bearing MDA-MB-231-tumors every 4 days, six times (Figure 6A). Monotherapy with paclitaxel or eribulin significantly inhibited tumor growth compared to that in the control group. The simultaneous administration of paclitaxel and eribulin significantly inhibited tumor growth compared to that with either monotherapy (paclitaxel group versus paclitaxel plus eribulin group, *p* < 0.01; eribulin group versus paclitaxel plus eribulin group, *p* < 0.01; Figure 6B). Body weight changes throughout treatment were not observed in any of the four groups (Figure 6C).

Furthermore, we performed immunohistochemical analysis using specimens obtained from this xenograft model. Compared to the tumors from control mice, the tumors from mice treated with eribulin showed significantly increased E-cadherin expression (*p* < 0.05) but significantly decreased vimentin expression (*p* < 0.05) at day 25 (Figure 6D, 6E). In contrast, the tumors from mice treated with paclitaxel showed significantly decreased E-cadherin expression (*p* < 0.01) but increased vimentin expression. The results were concordant with *in vitro* experiments, indicating that eribulin induces MET, whereas paclitaxel induces EMT, both *in vivo* and *in vitro*.

### Effects of pre-treatment with eribulin on anti-tumor activity of paclitaxel *in vivo*

**Figure 7:**
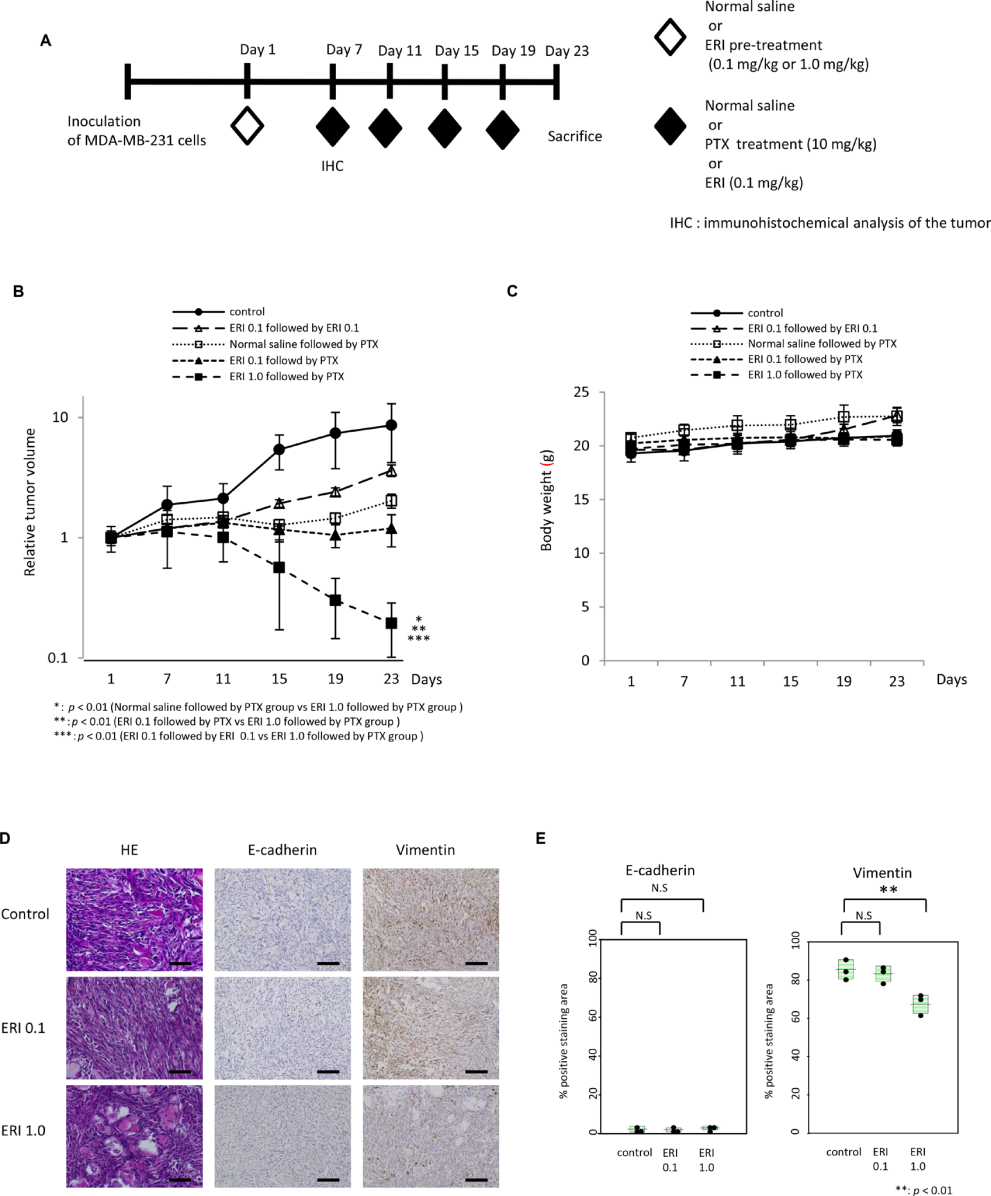
Effects of pre-treatment with eribulin on the anti-tumor activity of paclitaxel in MDA-MB-231 mouse xenograft models Anti-tumor effect of paclitaxel (PTX) with eribulin (ERI) pre-treatment was tested in an MDA-MB-231 tumor xenograft model. A single dose of 0.1 mg/kg or 1.0 mg/kg ERI or normal saline was administered to mice bearing MDA-MB-231-tumors 6 days before initiating treatment with the appropriate agents. Subsequently, 10 mg/kg PTX, 0.1 mg/kg ERI, or normal saline (control) was administered every 4 days, and mice were sacrificed on day 23. (**A**) Schematic representation of the experiment. (**B**) Average relative tumor volumes were plotted from day 1 to day 23 every 4 days. Closed circles (●) indicate control, closed triangles (▲) indicate 0.1 mg/kg of ERI (ERI 0.1) followed by PTX, closed squares (■) indicate 1.0 mg/kg of ERI (ERI 1.0) followed by PTX, open squares (□) indicate normal saline followed by PTX, and open triangles (○) indicate ERI 0.1 followed by ERI 0.1. Error bars represent standard deviation. ^*^p < 0.01 (normal saline followed by PTX group vs. ERI 1.0 followed by PTX group), ^**^p < 0.01 (ERI 0.1 followed by PTX vs. ERI 1.0 followed by PTX group), ^***^p < 0.01 (ERI 0.1 followed by ERI 0.1 vs. ERI 1.0 followed by PTX group). (**C**) The average body weight was measured from day 1 to day 23 every 4 days in each group. (**D**) Representative photographs of HE staining and immunohistochemistry (×400) for E-cadherin and vimentin in the tumors 6 days after administration of normal saline (control) or eribulin (0.1 mg/kg or 1.0 mg/kg). Each tumor was obtained from the xenograft models on day 7. Scale bars = 200 µm. (**E**) Comparison of the quantitative data of positive staining area for E-cadherin or vimentin observed in the three groups.

Next, we investigated whether pre-treatment with eribulin could enhance the anti-tumor effect of paclitaxel in an MDA-MB-231 tumor xenograft model. We evaluated the anti-tumor activity of 10 mg/kg paclitaxel with or without pre-treatment with eribulin in the MDA-MB-231 xenograft model. A single dose of eribulin (0.1 mg/kg or 1.0 mg/kg) was administered to mice bearing MDA-MB-231 tumors 6 days before the initiation of treatment with 10 mg/kg of paclitaxel, 0.1 mg/kg of eribulin, or normal saline (control; Figure 7A). Although a significant difference was not observed, a single administration of 0.1 mg/kg of eribulin followed by 10 mg/kg paclitaxel reduced the average tumor volume compared to that observed after treatment with normal saline followed by 10 mg/kg of paclitaxel or after treatment with eribulin followed by 0.1 mg/kg of eribulin. When the pre-treatment dose of eribulin was increased to 1.0 mg/kg, a single administration of 1.0 mg/kg eribulin significantly reduced the average tumor volume, compared to that in animals pre-treated with 0.1 mg/kg eribulin or saline (*p* < 0.01), and enhanced the anti-tumor activity of paclitaxel (Figure 7B). No body weight changes were observed throughout treatment in all the four groups (Figure 7C).

Furthermore, to investigate the induction of MET in cancer cells after a single administration of eribulin, we obtained tumor specimens from the xenografts on day 7 and examined the expression of E-cadherin and vimentin by immunohistochemical analysis. E-cadherin expression was not altered after treatment with eribulin, whereas vimentin expression decreased in a dose-dependent manner. Moreover, a significant decrease was observed in the tumors pre-treated with 1.0 mg/kg of eribulin compared to the control tumors (*p* < 0.01; Figure 7D, 7E). Thus, pre-treatment with eribulin induced MET in TNBC tumors in a mouse xenograft model and enhanced the anti-tumor activity of paclitaxel *in vivo* and *in vitro*.

## DISCUSSION

In the present study, we demonstrated a synergistic anti-tumor effect between eribulin and paclitaxel using MDA-MB-231 and Hs578T cells *in vitro*. Administration of low doses of eribulin or paclitaxel, which had little growth inhibitory effect alone, enhanced sensitivity to each other. Moreover, in an *in vivo* MDA-MB-231 xenograft model, administration of eribulin and paclitaxel, both in combination and sequentially, resulted in significantly enhanced anti-tumor effects, compared to the effect of monotherapy with either drug. To the best of our knowledge, this is the first report demonstrating a potential synergistic anti-tumor effect by combining two anti-tubulin agents, specifically eribulin and paclitaxel, for TNBC.

EMT plays a crucial role in the development of invasive and metastatic properties during cancer progression [24–26]. In addition to this role, EMT is associated with resistance to several cytotoxic drugs such as cisplatin, oxaliplatin, gemcitabine, 5-FU, paclitaxel, and an EGFR-tyrosine kinase inhibitor [27–34]. Paclitaxel and 5-FU have been reported to induce EMT directly in breast cancer cells [17, 22]. However, Wang *et al.* reported that tamoxifen induced MET in the mesenchymal TNBC cells [35]. In the present study, we showed that paclitaxel treatment increased the expression of mesenchymal markers in two TNBC cell lines, and this result was consistent with that observed in previous studies. In contrast, treating TNBC cells with eribulin decreased the expression of these mesenchymal markers, accompanied by increased expression of the epithelial marker; this was contrary to the phenotypic switch induced by paclitaxel. Recently, Yoshida *et al.* demonstrated that eribulin induces a phenotypic switch from a mesenchymal to an epithelial state in TNBC cell lines [21]. In the present study, a clear phenotypic switch from mesenchymal to epithelial state was induced by eribulin in MDA-MB-231 and Hs578T cells; however, this was not observed in Mx-1 and MDA-MB-157 cells. Hence, susceptibility of phenotype switches to eribulin is considered to depend on biological factors other than hormone receptors and HER2 in TNBC cells; however, the induction of MET by eribulin in a subset of TNBC cells represents a unique phenotypic change, opposite what has been reported for other cytotoxic agents [17, 22].

TGF-β is known to be the most potent inducer of EMT in many cell types. Recent studies have demonstrated that reversing EMT, by blocking TGF-β signaling with TGF-β type I receptor kinase inhibitors, enhances the anti-tumor effects of paclitaxel on TNBC and attenuates metastasis after treatment [17, 18]. As eribulin induced MET in MDA-MB-231 and Hs578T TNBC cells, we hypothesized that this drug might enhance the anti-tumor effect of paclitaxel by reversing EMT in these TNBC cells. As expected, both concurrent and sequential administration of low doses of eribulin significantly enhanced the anti-tumor effect of paclitaxel *in vivo* and *in vitro*. Moreover, even single administration of a low dose of eribulin, which also induced an epithelial phenotypic switch, significantly increased sensitivity to subsequent administration of paclitaxel both *in vitro* and *in vivo*.

Furthermore, TGF-β1, which induced EMT in MDA-MB-231 and Hs578T cells, enhanced sensitivity to eribulin in our study. In contrast, a forced transition to an epithelial phenotype by inhibition of ZEB1 by siRNA decreased sensitivity to eribulin in these cells. Dezzo *et al.* [36] reported that the expression of EMT-related genes is positively associated with eribulin sensitivity, and our present findings are consistent with this. In the present study, a single administration of low dose paclitaxel, which induced EMT in the cancer cells, significantly increased sensitivity to subsequently administered eribulin *in vitro*. Thus, the present and previous findings collectively indicate that a subset of TNBC cells become more sensitive to eribulin, when the cells change to mesenchymal phenotype; however, the precise mechanisms underlying the mesenchymal phenotype-induced increase in sensitivity to eribulin remains unknown. Our findings suggest that the cytotoxic effects of these anti-tubulin agents might partially depend on the current epithelial/mesenchymal status of the cancer cells in a subset of TNBC, although other mechanisms might also affect the cellular response to eribulin.

Although eribulin significantly improved OS without increasing PFS [10, 11], the underlying mechanisms are not completely clear. As a possible explanation for this clinical observation, Funahashi *et al.* demonstrated that eribulin-induced remodeling of abnormal tumor vasculature leads to a more functional microenvironment in MDA-MB-231 xenografts, and suggested that increased tumor perfusion might contribute to increased anti-tumor effects of subsequent treatments [37].

As another possible explanation, Terashima *et al.* suggested the possibility of a synergistic anti-tumor effect between eribulin and 5-FU through alterations to the MET-EMT axis [17, 22]. In addition, Yoshida *et al.* demonstrated that eribulin could suppress the incidence of new metastasis through the induction of MET [21]. Although they demonstrated that short-term exposure to eribulin induces MET in TNBC cell lines, the phenotype of TNBC cells with acquired resistance to eribulin due to long-term eribulin exposure has not yet been described. Our data demonstrate for the first time that a subset of TNBC cells that acquired resistance after long-term exposure to eribulin maintained an epithelial phenotype induced in the early phase of eribulin treatment; this implies that a phenotypic mesenchymal to epithelial switch might occur in clinical metastatic breast cancer treated with eribulin. Our results, together with the previous findings, suggest that eribulin-induced MET might enhance the anti-tumor effects of subsequently administered drugs, and that this might lead to increased OS in patients with metastatic breast cancer.

TNBC represents a highly diverse subset of cancer. Lehmann *et al.* categorized TNBC into six subtypes based on genetic expression profiling as follows: basal-like 1, basal-like 2, immunomodulatory, mesenchymal, mesenchymal stem-like, and luminal androgen receptor subtype [38]. According to this classification, MDA-MB-231, Hs578T, and MDA-MB-157 cells are classified into the mesenchymal stem-like subtype, which is enriched in genes involved in cell differentiation pathways (e.g., Wnt pathway, anaplastic lymphoma kinase pathway, and TGF-β pathway) [38]. In contrast, Mx-1 cells have a BRCA1 deletion (BRCA1 22626delGAAA) [39–41], and should be classified as basal-like subtype. In our study, eribulin and paclitaxel were shown to have a synergistic anti-tumor effect in MDA-MB-231 and Hs578T cells, but not in MDA-MB-157 and Mx-1 cells; this difference cannot be explained by the subtype differences described above. Thus, to predict subtypes of TNBC for which eribulin and paclitaxel will have a synergistic effect, the discovery of other molecular markers is required. A synergistic anti-tumor effect with eribulin has been reported for 5-FU previously and for paclitaxel in the present study; however, there have been no reports for other cytotoxic agents. More intensive studies are warranted to elucidate the molecular mechanisms underlying this synergistic action.

In conclusion, further studies are required to elucidate the molecular mechanisms underlying the MET-EMT phenotypic switch caused by eribulin as well as paclitaxel; however, our present study suggests the possibility of a novel therapeutic strategy, specifically combining two existing anti-tubulin agents, for a subset of TNBC. Moreover, it will be possible to optimize treatment by selecting a subset of TNBC, similar to MDA-MB-231 or Hs578T, that will respond to this therapeutic strategy. Future studies will be required to identify molecular markers that can predict responsiveness to combinatory anti-tubulin regimens.

## MATERIALS AND METHODS

### Cell culture and agents

Three TNBC cell lines (MDA-MB-231, Hs578T, and MDA-MB-157) were purchased from the American Type Cell Collection (Manassas, VA), and Mx-1 cells were purchased from CLS Cell Lines Service (Eppelheim, Germany) in 2016, and passaged in our laboratory for less than 6 months after receipt or resuscitation. All cell lines were tested monthly for mycoplasma contamination using the MycoAlert mycoplasma detection kit (Lonza Walkersville, Inc, Walkersville, MD) and were cultured for no more than 20 passages from the validated stocks. All cell lines were cultured in RPMI with 10% FBS at 37.0° C and 5% CO_2_. Eribulin-resistant MDA-MB-231 and Hs578T cells were previously established in our laboratory [23].

Eribulin was purchased from Eisai Co., Ltd. (Tokyo, Japan). Paclitaxel for *in vitro* use was purchased from Sigma-Aldrich (Saint Louis, MO) and for *in vivo* use was purchased from Nippon Kayaku Co., Ltd. (Tokyo, Japan). Recombinant human TGF-β1 (TGF-β) was purchased from R&D Systems (Minneapolis, MN). TGF-β receptor I kinase inhibitor (LY2157299) was purchased from Sellek Chemicals (Houston, TX).

### WST assays

The growth inhibitory effects of eribulin and paclitaxel were quantitated using a tetrazolium salt-based proliferation assay (WST assay; Wako Chemicals, Osaka, Japan) according to the manufacturer’s instructions. Briefly, 4 × 10^3^ cells were cultured in 96-well plates, in triplicate, with 100 μl of growth medium with a graded concentration of eribulin or paclitaxel for 96 h. Subsequently, 10 μl of WST-8 solution was added to each well, and the plates were incubated at 37° C for another 3 h. Absorbance was measured at 450 and 640 nm using the SoftMax Pro (Molecular Devices, Tokyo, Japan), and cell viability was determined. Each experiment was independently performed at least three times. The media supplemented with 10% FBS were used in the assays, except for the assays performed for testing the effect of TGF-β1 or a selective TGF-β type I receptor kinase inhibitor, LY2157299. In these assays, media containing 1% FBS were used.

### Combinatory effect of eribulin and paclitaxel *in vitro*

The effect of eribulin and paclitaxel in combination was measured using a WST assay, as mentioned previously. Here, 4 × 10^3^ cells were cultured for 96 h in 96-well plates with 100 μl of growth medium containing a graded concentration of eribulin and a low concentration of paclitaxel or growth medium containing a graded concentration of paclitaxel and a low concentration of eribulin. To evaluate the synergistic effect of paclitaxel and eribulin, an isobologram [42] was plotted based on data from the WST assay. In an isobologram, a diagonal line represents an additive effect. Experimental data points, represented by dots located below, on, or above the line indicate synergistic, additive, or antagonistic effects, respectively.

### Western blotting

Proteins were isolated from cells, as previously described, and were then used for western blot analyses (10 µg/lane) [43]. In the experiments with drug exposure, the proteins were isolated from cells treated with drugs for 48 or 96 h. The membrane was probed with the following antibodies: anti-pSmad2 (1:1000; Cell Signaling Technology, Danvers, MA), anti-ZEB1 (1:1000; Cell Signaling Technology), anti-E cadherin (1:50000, Gene Tax, Irvine, CA), anti-Slug (1:1000; Cell Signaling Technology), and anti-vimentin (1:1000; Cell Signaling Technology). β-Actin (1:5000; Sigma-Aldrich) was used as a loading control. Each experiment was performed independently at least three times, and one representative blot was chosen for the figures.

### Small interfering RNA (siRNA) transfection

ON-TARGETplus siRNA for ZEB1 (M-006564) and the negative control (D-001810) were purchased from GE Healthcare (Buckinghamshire, UK). Transfection of each siRNA (10 nM) was performed using Lipofectamine RNAi-MAX (Thermo Fisher Scientific, Waltham, MA) following the manufacturer’s instructions. Twenty-four hours after transfection, the proteins were extracted and 4 × 10^3^ cells/well were cultured in 96-well tissue culture plates and incubated for 72 h after adding stepwise diluted eribulin. Finally, the absorbance was measured after adding the WST solution, as described previously.

### Cell growth assay

The growth of parental and eribulin-resistant MDA-MB-231 and Hs578T cells was measured by performing a WST assay (Wako Chemicals). Briefly, 4 × 10^3^ cells/well were cultured in 96-well plates in 100 µl medium. After each indicated period, the absorbance was measured after adding WST solution, as described previously. Each experiment was independently performed at least three times.

### Wound healing assay

Cells were seeded in culture inserts (Ibidi, Martinsried, Germany). Inserts were removed when cells reached 90% confluency and medium was replaced. Subsequently, cells were incubated for 12 h (Hs578T cells) or 24 h (MDA-MB-231 cells). Images of cells were captured with a camera attached to a microscope (KEYENCE, Osaka, Japan). The proportion of wound closure at 0 h and 12 h or 24 h was measured using 100 random points. The average of wound closure was calculated as a ratio of the initial wound size.

### Experimental mouse model for combination treatment of eribulin and paclitaxel

Five-week-old female BALB/c-nu/nu mice (Charles River Laboratories Japan, Inc., Yokohama, Japan) were used for *in vivo* studies. All animal experimental procedures were conducted based on protocols preapproved by the Institutional Animal Care and Use Committee of Shinshu University. MDA-MB-231 cells (8 × 10^6^) with PBS containing 50% Matrigel were injected subcutaneously. To test the effect of concurrent administration of eribulin and paclitaxel, tumor-bearing mice were divided randomly into four groups (*n* = 3 per group), when tumor volume was approximately 60–100 mm^3^. Each group of mice was intraperitoneally administered normal saline, eribulin (0.1 mg/kg), paclitaxel (10 mg/kg), or both at the same time every 4 days, six times. Mouse weight was determined every 4 days. Tumor diameters were measured with calipers, and tumor volume was calculated using the following formula: volume = (length/2) × width^2^. Relative tumor volume was calculated using the following formula: tumor volume at the measuring day/tumor volume at day 1.

To test the effects of eribulin pre-treatment on the anti-tumor activity of paclitaxel, tumor-bearing mice were divided randomly into five groups (*n* = 3 per group). Mice were pre-treated with intraperitoneal administration of normal saline or eribulin once on Day 1. Two groups of mice were administered normal saline, other two groups were administered 0.1 mg/kg of eribulin, and one group was administered 1.0 mg/kg of eribulin. Six days later, we initiated the administration of normal saline, paclitaxel (10 mg/kg), or eribulin (0.1 mg/day) every 4 days, four times. Normal saline was administered to a group pre-treated with normal saline; this group was designated as the control group. Paclitaxel (10 mg/kg) was administered to the other three groups pre-treated with normal saline, 0.1 mg/kg of eribulin, or 1.0 mg/kg of eribulin; these groups were designated as normal saline followed by PTX, ERI 0.1 followed by PTX, and ERI 1.0 followed by PTX, respectively. ERI (0.1 mg/kg) was administered to a group pre-treated with 0.1 mg/kg of ERI, and this group was designated as ERI 0.1 followed by ERI 0.1. Relative tumor volume was calculated using the previously mentioned formula.

### Immunohistochemistry

Sections (3-μm) of paraffin-embedded tumor samples were used for HE staining and immunohistochemistry. The following antibodies and dilutions were used: anti-E-cadherin (1:500, Gene Tax) and anti-vimentin (1:100; Cell Signaling Technology). Immunohistochemical staining was performed as previously described [43]. The expression of E-cadherin and vimentin was quantitated based on the percentage of positive staining area in each sample by using Keyence BZ-II software (KEYENCE, Osaka, Japan). The averages of positive staining ratios were calculated from five randomly selected areas in each sample.

### Statistical analysis

Data were tested for significance by performing unpaired Student’s *t*-tests; a *p*-value of < 0.05 was considered statistically significant (StatFlex ver.6, Artech Co., Ltd., Osaka, Japan).

## SUPPLEMENTARY MATERIALS FIGURES AND TABLE


