# Supplementary material for: Combination of two anti-tubulin agents, eribulin and paclitaxel, enhances anti-tumor effects on triple-negative breast cancer through mesenchymal-epithelial transition
Source: Oncotarget. 2018 May 1;9(33):22986–3002. doi: 10.18632/oncotarget.25184 (PMC5955406; doi:10.18632/oncotarget.25184)
Supplement: Supplementary file 1 [file oncotarget-09-22986-s001.pdf]

Combination of two anti-tubulin agents, eribulin and paclitaxel, enhances anti-tumor effects on triple-negative breast cancer through mesenchymal-epithelial transition

SUPPLEMENTARY MATERIALS

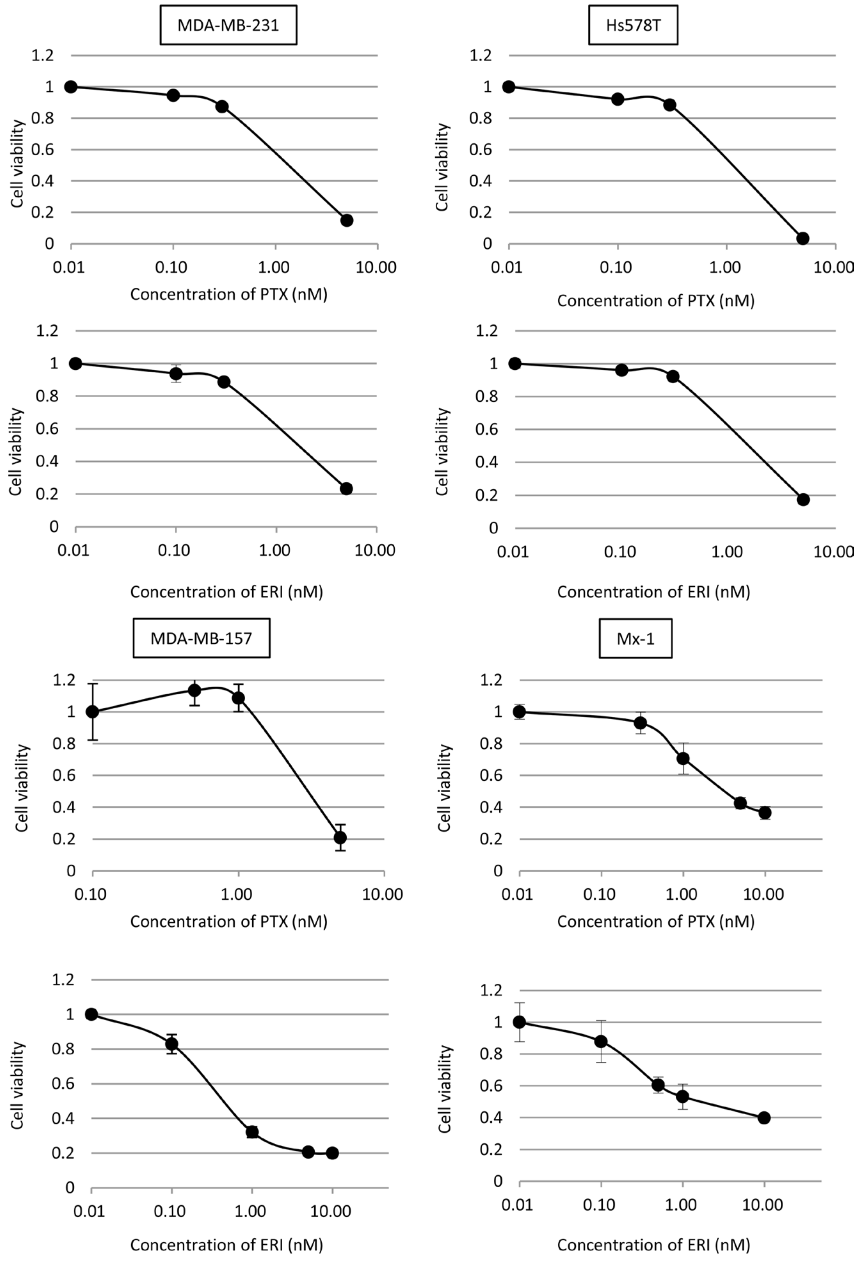

Supplementary Figure 1: Growth inhibitory effect of eribulin and paclitaxel in triple-negative breast cancer (TNBC) cell lines. The growth inhibitory effect of eribulin and paclitaxel was tested in four TNBC cell lines (MDA-MB-231, Hs578T, MDA-MB-157, and Mx-1), and the concentrations at which cell growth was not inhibited were determined for each drug and each cell line.

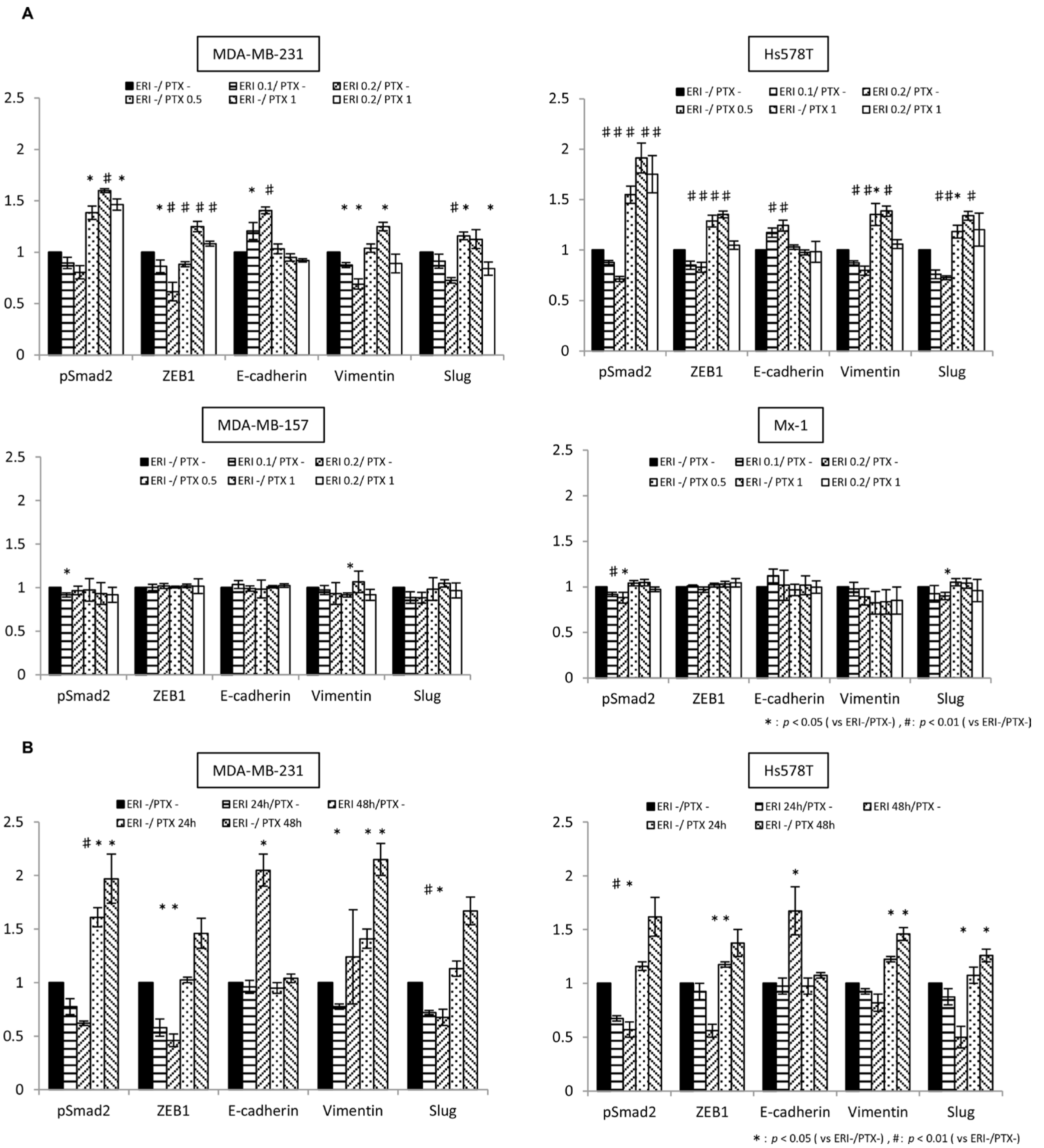

Supplementary Figure 2: Expression of epithelial/mesenchymal markers in triple-negative breast cancer (TNBC) cell lines. The expression of epithelial and mesenchymal markers was studied by western blotting. The expression of proteins of interest was normalized with that of β-actin, used as an internal control in each experiment. Histograms represent the average and SD of relative protein expression calculated from three independent experiments. (**A**) Expression of epithelial-mesenchymal transition (EMT) markers in MDA-MB-231, Hs578T, MDA-MB-157, and Mx-1 cells treated with eribulin (ERI; 0.1 and 0.2 nM), paclitaxel (PTX; 0.5 and 1 nM), or both (ERI; 0.2 nM and PTX; 1 nM) for 96 h. (**B**) Expression of EMT markers in MDA-MB-231 and Hs578T cells treated with ERI (0.2 nM) or PTX (1 nM) for 24 h and 48 h. $^{*}p < 0.05$ compared to untreated (ERI^{-}/PTX^{-}) cells and $^{\#}p < 0.01$ compared to untreated (ERI^{-}/PTX^{-}) cells.

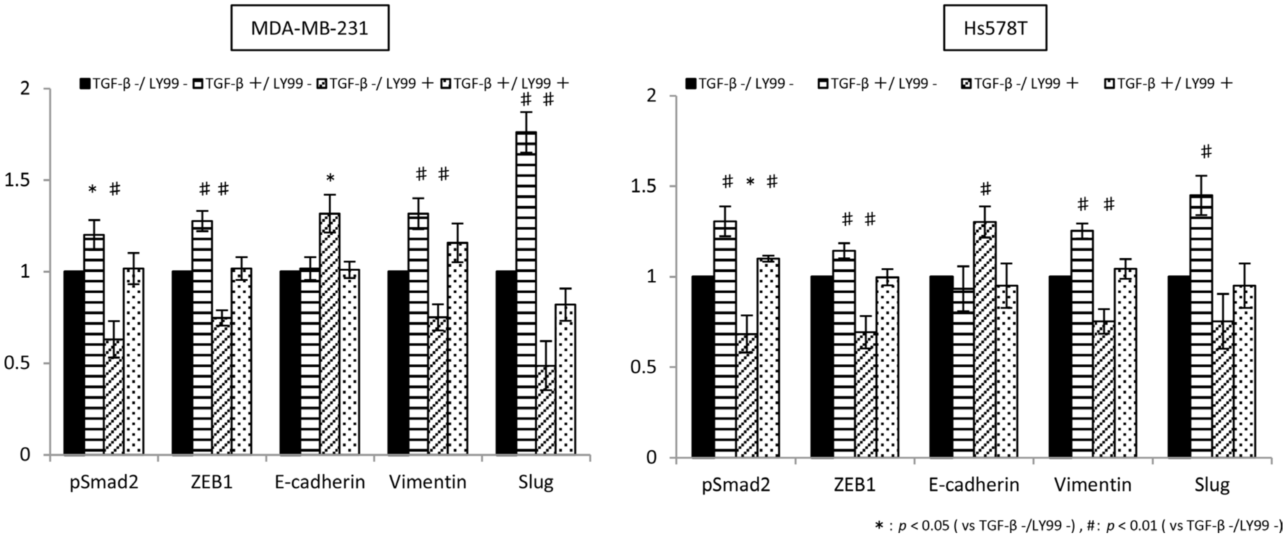

Supplementary Figure 3: Effects of TGF-β or TGF-β type I receptor kinase inhibitor on the expression of epithelial/ mesenchymal markers in MDA-MB-231 and Hs578T cells. MDA-MB-231 and Hs578T cells were pre-treated with TGF-β (10 ng/ml) or the TGF-β type I receptor kinase inhibitor LY2157299 (5 µM), or both for 2 days. Then, the expression of epithelial/mesenchymal markers was studied by western blotting. The expression of proteins of interest was normalized with that of β-actin, used as an internal control in each experiment. Histograms represent the average and SD of relative protein expression calculated from three independent experiments. *$p < 0.05$ compared to untreated (TGF-β⁻/LY99⁻) cells and #$p < 0.01$ compared to untreated (TGF-β⁻/LY99⁻) cells.

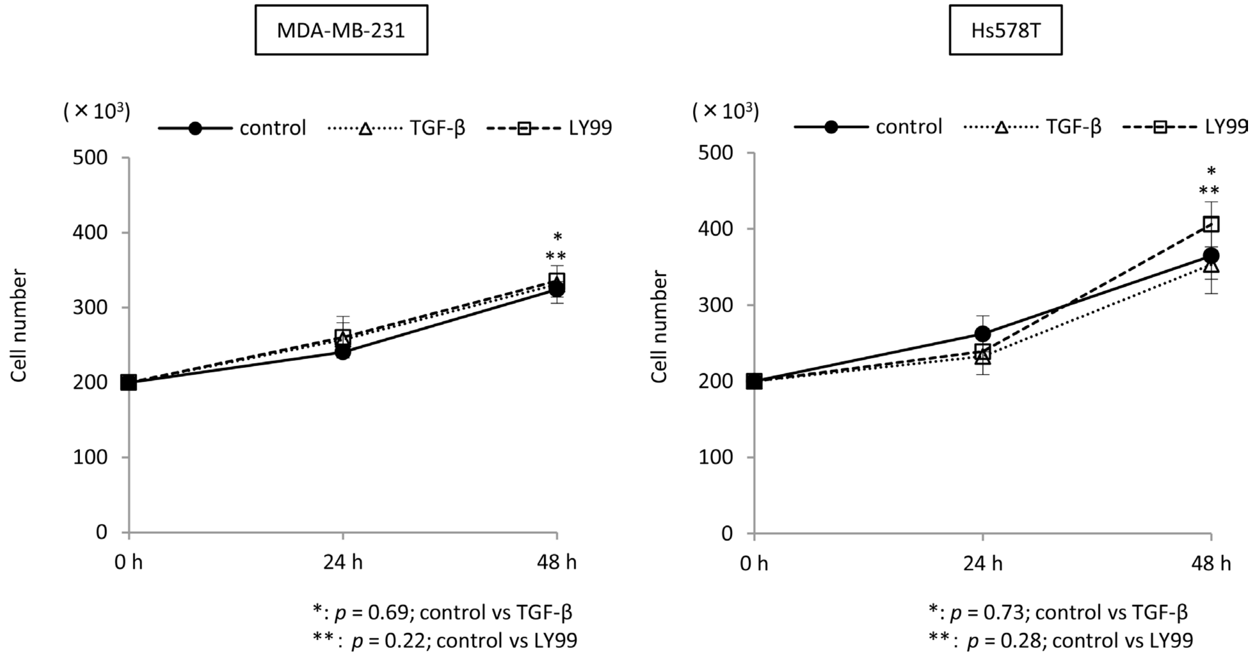

Supplementary Figure 4: Proliferation of MDA-MB-231 and Hs578T cells after addition of TGF-β1 or LY2157299. The effect of TGF-β1 (10 ng/ml) and LY2157299 (LY99; 5 µM) on proliferation of MDA-MB-231 and Hs578T was tested. The cells were counted at 24 h and 48 h after seeding. Both TGF-β1 and LY2157299 did not affect the proliferation of two TNBC cell lines.

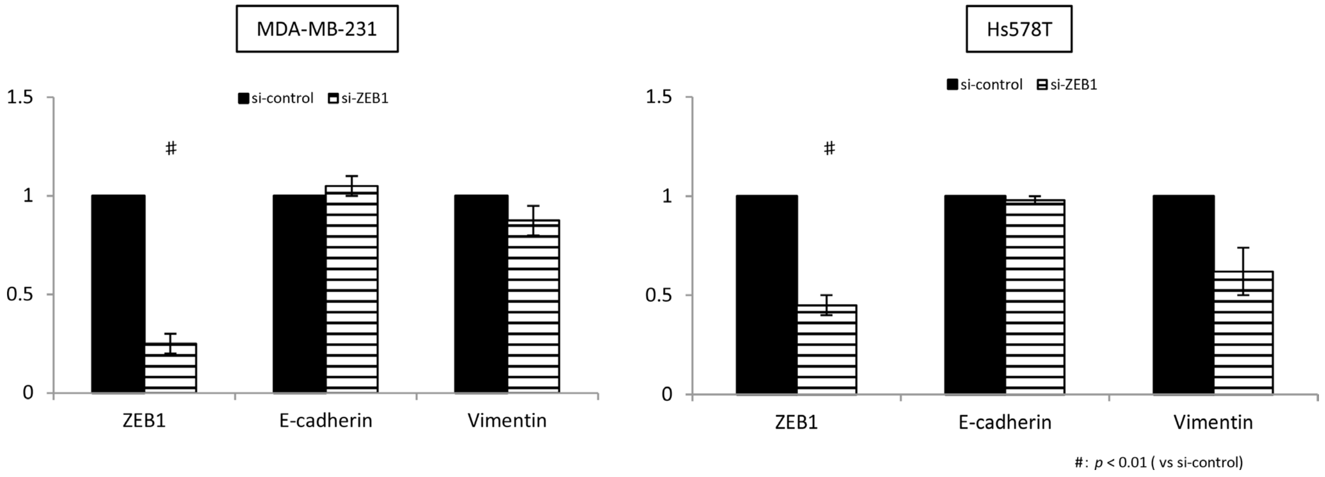

Supplementary Figure 5: Effects of ZEB1 inhibition on the expression of epithelial/mesenchymal markers in MDA-MB-231 and Hs578T cells. The expression of ZEB1 was inhibited by siRNA, and protein was extracted 24 h after transfection. The expression of ZEB1, E-cadherin, and vimentin was analyzed by western blotting. The expression of proteins of interest was normalized with that of β-actin, used as an internal control in each experiment. Histograms represent the average and SD of relative protein expression calculated from three independent experiments. $^{\#}p < 0.01$ compared to si-control.

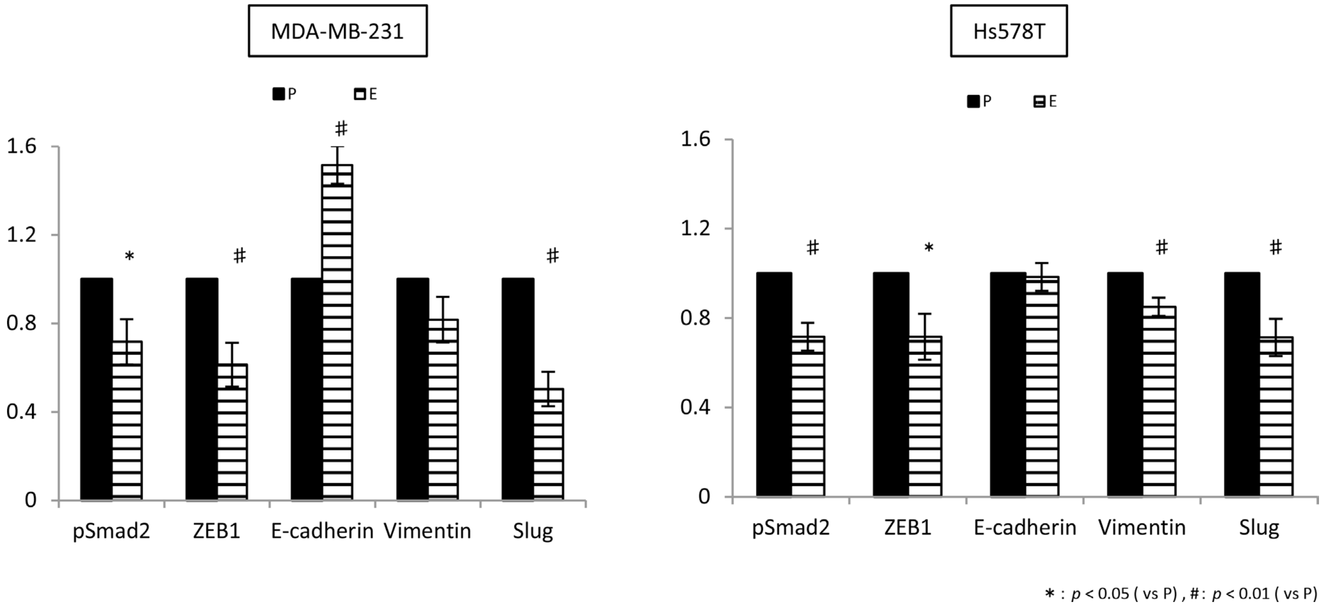

Supplementary Figure 6: Expression of epithelial/mesenchymal markers in eribulin-resistant MDA-MB-231 and Hs578T cells. The expression of pSmad2, ZEB1, E-cadherin, vimentin, and Slug in eribulin-resistant MDA-MB-231 and Hs578T cells and the respective parental cells was analyzed by western blotting. The expression of proteins of interest was normalized with that of β-actin used as an internal control in each experiment. Histograms represent the average and SD of relative protein expression calculated from three independent experiments. $^{*}p < 0.05$ compared to parent cells and $^{\#}p < 0.01$ compared to parent cells.

Supplementary Table 1: IC_{50} for eribulin and paclitaxel in triple-negative breast cancer cell lines

Cell line	IC_{50} of eribulin (nM)	IC_{50} of paclitaxel (nM)
MDA-MB-231	1.2 ± 0.3	1.1 ± 0.1
Hs578T	1.1 ± 0.1	1.1 ± 0.3
MDA-MB-157	0.8 ± 0.1	2.8 ± 0.4
Mx-1	1.0 ± 0.2	1.0 ± 0.1

IC_{50}: half-maximal inhibitory concentration.
